# India’s Bhilwara COVID-19 containment policy response: Lessons for public health policy makers globally

**DOI:** 10.7189/jogh.10.020325

**Published:** 2020-12

**Authors:** Mahaveer Golechha

**Affiliations:** Health Systems and Health Policy, Indian Institute of Public Health-Gandhinagar, India

The coronavirus disease 19 (COVID-19) pandemic is unique and unprecedented in several aspects and has challenged health care systems across the globe. Over the past few weeks political leaders, policymakers, and public health managers across the globe grapple with the reality of a novel coronavirus outbreak and looking for potential solutions for containment of COVID-19.

Bhilwara district of Rajasthan, a western Indian state has shown a way to public health policymakers globally for containing COVID-19 with effective screening and containment strategy combined with a stringent lockdown. The Corona Containment policy response of Bhilwara had made it only district in the country with initial such high number of cases to a rapid decline in COVID-19 patients in a short span of 20 days. The ‘Bhilwara model’ has been so successful in containing the COVID-19 and become an example for other Indian states and policymakers.

The Bhilwara with aggressive early contact tracing and extensive surveillance, rational testing, effective lockdown, efficient risk communication and community engagement, adequate quartile and isolation along with decentralisation of authority seem to offer examples of successful containment to public health policymakers across the globe.

China, Taiwan, South Korea and Japan and some European nations with highly sophisticated well-resourced health systems are also successful in containing COVID-19, however, their plans may not be feasible for other countries due to resource limitation. But Bhilwara model can, as it has shown the containment with limited resources. Countries such as South Korea isolated infected people based on widespread testing, but Bhilwara’s mass-surveillance approach has achieved a similar goal, and be relevant for other low- and middle-income countries facing scarcity of resources.

Healthcare systems across developed and developing nations are being put to the ultimate test and are under tremendous pressure to limit the spread of the novel coronavirus [1]. The emergence of SARS-CoV-2 and the menace of imminent endemics have brought into forefront the urgent need to prepare for the consequences of associated epidemics and pandemics [2]. Unlike the “Spanish flu” of 1918, which became an international epidemic over the course of a year, COVID-19 has spread to every inhabitable continent within weeks, outpacing our health system’s ability to test, track, and contain people with suspected infection [3]. Over the past few weeks political leaders, policymakers, and public health managers across the globe grapple with the reality of a novel coronavirus outbreak and looking for potential solutions for containment of COVID-19 [4].

Bhilwara with its effective screening and containment strategy combined with a stringent lockdown flatten the curve in a short span of 3 weeks. The district administration called the strategy as ruthless COVID-19 containment plan. The ‘Bhilwara model’ has been so successful in curbing the spread of COVID-19 in a hotspot that the central government has asked other Indian states to replicate its model of “ruthless containment”.

Bhilwara district is one of the four districts, those comes under Ajmer division of state of Rajasthan. District Collector and District Magistrate is the head of District Administration. The district is divided in 12 sub-divisions and tehsils (Sub-Districts) with 1834 villages. As per recent data the population of Bhilwara district is 2 704 384 with 78.7 percent rural and 21.3 percent urban population. The district has a total area of 10 455 km^2^, 311 km^2^ is urban and 10 144 km^2^ is rural [5].

The district was among the handful of districts in the country where the coronavirus pandemic threatened to spiral out of control, with two dozen positive cases recorded in the first few days. Worryingly, the first positive case reported on 18 March in the city was a medical doctor of a private hospital and within a few days’ the district witnessed more than two dozen positive cases. The doctor was running a hospital and was also a visiting consultant at several other health facilities. The doctor was asymptomatic and along with others carried a risk of having met thousands of patients who had visited the hospital during a fortnight or more. Hospital records indicated that around 7000 patients came in direct contact with the first infected medical doctor were belongs to 15 districts of states and 4 neighbouring states. A line list of high risk contacts was prepared, consisting of 613 in-patients, 96 ICU patients, 5600 outpatients of Bhilwara district, 36 outpatients of 4 neighbouring states, and 498 patients of 15 districts of Rajasthan. This made the task of authorities very difficult for containing the spread of the virus and halting community transmission. Initially, the district seems to become Lombardy of India. All the cases confirmed so far are those identified through contact tracing of the patients and staff that came into the contact with the first doctor who was found infected.

Bhilwara had a surge of COVID-19 outbreak and the virus started spreading at the local and community level. The number of new coronavirus cases increases exponentially peaking at 26 new infections on March 30. But the new case has dropped significantly and the district has reported only 10 new cases in the last 4 weeks ending on 4 May 2020 [6], and 32 out of 37 patients have been recovered. Bhilwara was able to successfully flatten the curve on COVID-19 in only 20 days with its containment strategy executed with clockwork precision, immaculate coordination, and extreme efficiency.

The Bhilwara containment strategy was largely based on WHO’s pillars of the public health response for COVID-19, suggested in its Operational Planning Guidelines to support country preparedness and response [7].

The Bhilwara containment policy response involves following basic but crucial steps:

**Figure:**
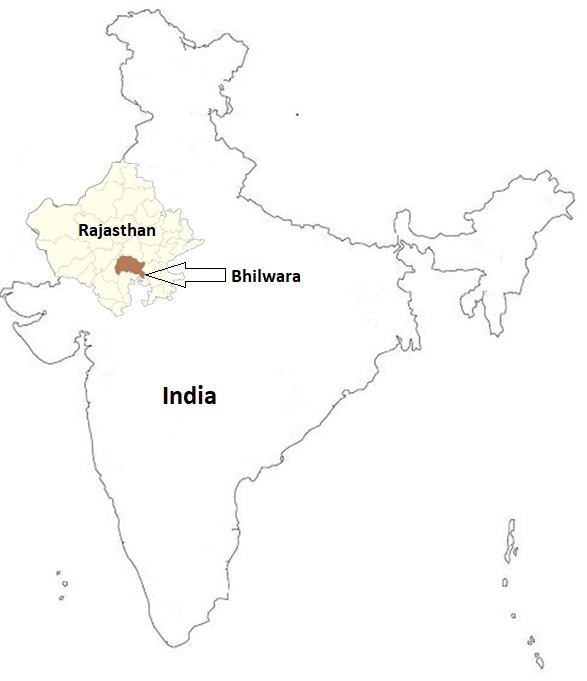
Photo: A map of India Map of India showing Rajasthan and Bhilwara District (source: https://d-maps.com/carte.php?num_car=24853&lang=en).

**Stringent and effective lockdown:** The district imposed a curfew (stringent lockdown) under Section 144 of the Code of Criminal Procedure (making gatherings of five or more persons unlawful) on 20 March 2020, five days before the nationwide lockdown. The district borders were sealed instantaneously, with 24 check-posts set up at all entry/exit points to monitor movement control. Further, the neighbouring district authorities also instructed to seal their borders with Bhilwara. All forms of transportation services and movement of vehicles were stopped, industries, shops, malls, and markets across the district were shut down with immediate effect. Several studies have also shown the decline in transmission due to control measures and restrictions during epidemic [8].

**Governance and multi-sectoral coordination:** The state government has acted swiftly and coordinated effectively from state headquarter. Several public health teams and technical experts deployed to the district with continuous monitoring by state government leadership including the chief minister, health minister, and senior bureaucrats. All the required resources have been made available to the district administration and the district administration was totally empowered to carry out containment measures optimally, without any interference from any quarter. The Epidemic Act was imposed immediately for giving the district administrator all the powers to take over hospitals, hotels, and other buildings. Putting up required legal measures are essential measures for supporting control. Around the clock war room was established at district headquarter, manned by three teams working in eight-hour shifts every day. They were tasked with monitoring every aspect of tracing, testing, quarantine, and lockdown in the district.

Transdisciplinary and multi-sectoral strategies are necessary for solving complex problems that threaten public health and safety [9]. Outbreak response requires coordination at all levels. The Bhilwara approach is truly multi-sectoral in nature, the district administration has coordinated with all line departments including the public health department, police, home guard, local municipal corporation, politicians, state disaster response force, private hospitals, community leaders, supplier, and religious leaders. This multisectoral approach has ensured the effectiveness of lockdown.

**Cluster mapping and contact tracing:** Intensified case finding and contact tracing are considered crucial by most nations and are being undertaken to attempt to locate cases and to stop onward transmission [10]. The administration has implemented the cluster containment plan. The faster the infected individuals are identified and quarantined, the lesser the number of future contacts. The district immediately mapped clusters within, ensuring targeted containment strategies were put in place to keep spread in check. After lockdown and isolation of the district, health teams started cluster mapping for identifying positive cases and tracing of their close contacts. Contact-tracing was a rigorous but crucial exercise that authorities have done to the place, quarantine, or treat possible patients. Following this exercise, six areas were identified and special teams were deployed for continuous screening of suspected cases. The buffer zones have been created for more focussed containment and swift tracing of contacts of primary patients and secondary contacts. The police and health team reached out to around 7000 people who were consulted by the COVID-19 positive doctor in just 2 days and all of them put into quarantine either at their own homes or at available facilities for breaking the chain of transmission.

**Robust surveillance and screening:** Due to limited resources available for testing, the Bhilwara model adopted mass surveillance and rigorous screening measures. Immediately, after lockdown 332 teams of health workers, police personnel, and volunteers been formed for the screening of the urban population, and similarly around 1900 teams have been deployed for screening and surveillance of the rural population of the district (**Table 1**). A rigorous screening strategy has been adopted, prioritizing the affected epicentres, as well as the migratory population. Teams were given clearly demarcated areas for conducting surveys. One supervisor has been appointed for every 10 survey teams. The screening has been carried out in gradual phases, the first phase started on 21 March, which includes door-to-door survey and medical screening of the entire population for identifying influenza-like-symptoms individuals, international travellers, and primary and secondary contacts of positive patients. The first phase of the survey completed on 28 March and teams have identified more than 19 000 people with influenza-like-symptoms. All of them have been sent to quarantine. The list has complied with address and other details of all symptomatic people displaying signs of cough, fever, cold and breathing problems.

**Table 1:** Surveillance and screening of district population by teams in various phases

	Teams	Household surveyed	Individual surveyed	Patients identified with influenza like symptoms
URBAN				
**First phase** (18-24 March)	1135	73 334	365 605	2572
**Second phase** (24 March-2 April)	1099	75 340	366 449	822
**Third phase** (27 March-3 April)	830	65 973	339 261	864
**RURAL**				
22 March-2 April	1937	441 953	2 239 134	17 597

In the second phase, a second survey and screening exercise of all those identified in the first list along with their family members has been carried out. This screening coordinated in every village by local government heads, block development officers, and block-level medical officers. Within 24 hours the screening exercise of the first list suspects and their family members was completed. The teams have been continuously following up on these cases, twice a day, to understand if they are exhibiting symptoms and need further treatment. These teams were reporting directly to Additional District Magistrate, some individuals are designated as Corona Fighters and some as Corona Captains. The names were given to ignite a feeling of war as the entire administration was fighting COVID-19 on a war footing.

These teams also ensured that people who were asked to self-quarantine are not venturing out of their homes. These teams constantly monitored quarantined families and sent daily reports on their health and isolation status to district authorities. The teams have carried out this mammoth task of screening a population of more than 2.35 million.

**Ramping up of Quarantine, Isolation, and treatment facilities:** Simultaneously, the district administration was building the infrastructure to meet the consequences of community transmission. The health infrastructure is ramped up in district hospital to expand its bed capacity from existing 200 beds to 427 beds (**Figure 1**). The capacity building of health care professionals been carried out to meet the clinical treatment and management of COVID-19 cases by state and district Rapid Response Teams (RRTs). The training of ground-level corona fighters was done by Sub-Divisional Magistrate, Block Development Officers and Chief Medical Health Officer through video conferencing. The district administration then took the unprecedented step of taking control of four private hospitals along with their staff and medical equipment with an isolation ward capacity of 25 each. To meet the requirement of quarantine facility to meet an emergency situation, rooms in hotels, resorts, and charitable trust guest houses have been acquired and altogether around 1500 quarantine beds. Furthermore, institutions and hostels are acquired and converted in to quarantine facilities with capacity of 11 659 beds for accommodating asymptomatic and patients with mild symptoms (**Figure 1**).

**Figure 1:**
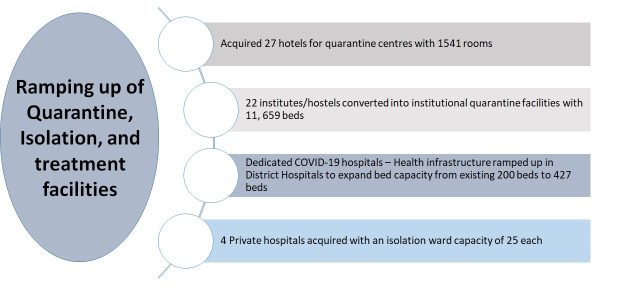
Visual representation of quarantine, isolation and treatment facilities.

The administration has carried out, a special sanitation drive and disinfection campaign in the entire district with the help of local municipal corporations. The teams were disinfecting every nook and corner of the district for containing the transmission-from all containment and buffer zones, to localities where positive cases have been detected, to all ambulances and police vehicles, screening centres, and quarantine centres, the collectorate, police stations and other public offices.

**Risk communication and community engagement with the humanistic approach:** Risk communication and community engagement is a critical component of the response to COVID-19 [7]. The administration has ensured that people have the right information, delivered in the right way, to take appropriate and proportionate steps to protect themselves. The communication mechanism helps people make the right decisions about how to protect themselves when to seek care, and to avoid contributing to panic about the disease and its effects. The administration has made effective use of ICT in community awareness. The district produces daily updates on epidemic progress for the public and reports for authorities, aiming to disseminate the most robust and reliable science to inform decision making. The administration has partnered with community and religious leaders to motivate the public to stay home. This approach has generated significant community capacity to respond to resiliently during the coronavirus crisis. Sharing accurate information and best practices in real-time is also critical to help counter the rapid spread of false and sometimes dangerous disinformation.

To reduce discomfort to the public due to stringent measures, the administration has ensured door-to-door regular supplies of milk, vegetables, grocery, and other essentials. For this, control rooms of several departments are being operated for fulfilling population demands. The administration has also worked together with NGOs for distributing packets of raw food as well as cooked food to the poor, vulnerable, migrant workers, and needy population. The officials in proactive collaboration with social workers, philanthropists, and industrialists arranged the food and supply of other essentials to those leading ‘a hand to mouth existence', truck drivers, street vendors, and daily wage workers. At the same time, around 3900 ‘helpless and homeless people’ in urban and rural areas were identified and provided shelter and food. The humanistic approach has prevented social unrest.

## CONCLUSION

Many countries with well-resourced health systems, like South Korea, Japan, New Zealand have also successfully contained the COVID-19, however, their strategy may not be feasible for resource-limited settings. However, Bhilwara model can be implemented in resource-limited countries. The success or failure of the fight against the COVID-19 pandemic will naturally be a measure of the overall capacity of health systems and administration and will have great implications for public health policymakers. I hope this perspective will be a small but meaningful policy guide for the international community and especially global health policymakers by sharing Bhilwara’s experiences and countermeasures against COVID-19.
